# A Fine-Grained Recognition Neural Network with High-Order Feature Maps via Graph-Based Embedding for Natural Bird Diversity Conservation

**DOI:** 10.3390/ijerph20064924

**Published:** 2023-03-10

**Authors:** Xin Xu, Cheng-Cai Yang, Yang Xiao, Jian-Lei Kong

**Affiliations:** 1 School of Artificial Intelligence, Beijing Technology and Business University, Beijing 100048, China; 2 State Environmental Protection Key Laboratory of Food Chain Pollution Control, Beijing Technology and Business University, Beijing 100048, China

**Keywords:** fine-grained bird species recognition, deep learning neural networks, graphic-related high-order embedding, ecological environment security, biodiversity conservation

## Abstract

The conservation of avian diversity plays a critical role in maintaining ecological balance and ecosystem function, as well as having a profound impact on human survival and livelihood. With species’ continuous and rapid decline, information and intelligent technology have provided innovative knowledge about how functional biological diversity interacts with environmental changes. Especially in complex natural scenes, identifying bird species with a real-time and accurate pattern is vital to protect the ecological environment and maintain biodiversity changes. Aiming at the fine-grained problem in bird image recognition, this paper proposes a fine-grained detection neural network based on optimizing the YOLOV5 structure via a graph pyramid attention convolution operation. Firstly, the Cross Stage Partial (CSP) structure is introduced to a brand-new backbone classification network (GPA-Net) for significantly reducing the whole model’s parameters. Then, the graph pyramid structure is applied to learn the bird image features of different scales, which enhances the fine-grained learning ability and embeds high-order features to reduce parameters. Thirdly, YOLOV5 with the soft non-maximum suppression (NMS) strategy is adopted to design the detector composition, improving the detection capability for small targets. Detailed experiments demonstrated that the proposed model achieves better or equivalent accuracy results, over-performing current advanced models in bird species identification, and is more stable and suitable for practical applications in biodiversity conservation.

## 1. Introduction

Avian diversity refers to the variety of bird species that exist in natural environments. Birds are one of the most diverse groups of vertebrate animals on earth and play various roles in ecosystems, such as pollination, occupying the top or bottom of the food chain, and controlling populations [1]. The conservation of avian diversity plays a critical role in maintaining ecological balance and ecosystem function, in addition to having a profound impact on human survival and livelihood. With factors such as environmental change, habitat loss, habitat destruction, and human activities, many bird species are facing the threat of endangerment and extinction on a global scale. Therefore, protecting and maintaining avian diversity has become a global priority, especially in complex outdoor environments, where the accurate identification of bird species is essential to protect ecological environment security and maintain changes in avian biodiversity [2,3].

From the defined scope, biodiversity refers to all plants, animals, and microorganisms on the earth and their genes. The ecosystem and its ecological process are formed by interacting with various species and their habitats, including intra-species, inter-species, and ecosystem diversity [4]. Monitoring biodiversity is not only an effective way to monitor the changes between organisms but also an effective way to evaluate their conservation effectiveness. It can provide an essential basis for formulating action plans and management measures related to biodiversity conservation [5,6]. Bird species diversity is an ecological combination formed by interacting with birds, other organisms, and the environment, e.g., the relationship between birds and air quality [7], and the impact between birds and wetland environments [8]. It can characterize the composition and structure of bird communities and the quality of bird habitats, which is of great significance for understanding the stability of ecosystems [9]. Meanwhile, birds have unique ecological and system engineering effects, such as species conservation and environmental restoration, and are an integral part of wetland ecosystems, which are relevant to the healthy development of human civilization [10]. Because birds have a broad niche and are sensitive to changes in ecological factors, they are biological indicator species for detecting environmental quality [11]. The species composition of birds reflects the comprehensive utilization of the bird habitat environment to a certain extent [12]. In addition, birds provide humans with important cultural connotations that inspire people to watch them, which is fascinating in the life history of birds. One example is bird watching, a sport that is very beneficial to human physical and mental health and that actually focuses on the diversity of birds [13].

In forestry and wetland ecosystems, bird identification technology can effectively help related staff to realize automatic bird monitoring and count the number and species distribution of birds in the natural ecological environment, which plays a decisive role in bird protection and has essential research value [14]. Traditional bird identification mainly relies on the experience of experts, some of whom specialize in bird work and research and some of whom use bird watching as a recreational activity. But in any case, these people still constitute a minority group [15]. Although this method can ensure high accuracy, it needs to rely on experts to identify birds manually, which is costly. Moreover, there are many kinds of wild birds, and their habitats are complex. It is inefficient and difficult to guarantee the accuracy rate only by manually interpreting and counting their types and distributions. In addition, humans cannot wait in the wild all the time, so how to effectively measure the existence and distribution of the bird population has always been a complex problem.

With the progress of computer and artificial intelligence technology, artificial intelligence technology has been gradually applied to various intelligent identification fields, e.g., intelligent health monitoring [16], agricultural hazard detection [17], remote sensing satellite image processing [18], pest identification [19], and food traceability [20]. Similarly, bird image recognition technology has made significant progress. In the early days, researchers mainly relied on pattern recognition by manually extracting classification features to recognize bird images [21]. Researchers must select key features of different birds, e.g., texture [22] and the gradient histogram [23], and then design corresponding digital feature vectors. By comparing these feature vectors, the classification and prediction of different bird targets can be realized. However, to minimize the error, it is often necessary to manually extract the features of crucial points and subtle differences, dramatically increasing the workload of manual feature extraction. Moreover, due to the different requirements of each task, researchers focus on selecting varying bird features, which weakens the generalization ability of this kind of bird classification method. It is difficult for the algorithm to be further popularized and applied [24], and it is also tricky to significantly improve its accuracy, bringing significant challenges to bird feature recognition [25].

With deep learning and convolutional neural network development, target classification and recognition technology have also made significant breakthroughs. Deep learning is a method to learn big data samples’ inherent laws and representation levels. In the learning process, much information can be obtained for data interpretation. Deep learning has been studied in many fields, e.g., natural language [26], images [27], and sounds [28]. Deep learning can replace much repetitive work performed by human beings with mighty computer computing power and dig deeper into the subtle feature differences among different birds with its deep feature extraction capability, which significantly improves classification accuracy. Deep learning image classification distinguishes different images according to their semantic information. The difference lies in that their features are not extracted or designed manually. Still, hierarchical feature learning is carried out supervised or unsupervised through deep neural networks. The pixel information of the images is directly used for input, which keeps all the knowledge of the input images to the greatest extent. Through convolution operation, feature extraction and high-level feature abstraction are carried out on the images, and its image classification effect has surpassed that of human beings under certain conditions. It has significant advantages in the field of bird recognition.

However, the bird data obtained in the virtual environment are often affected by the actual environmental factors, e.g., occlusion, light, and shadow, which significantly limits the performance of the bird recognition algorithm. Meanwhile, in the actual environment, there are fine-grained phenomena with minor interspecific differences and large species differences among birds, which are also essential factors restricting bird identification. Moreover, because the equipment used for bird detection is often fixed in the virtual environment, this leads to a situation in which the target in the obtained bird image is tiny, which leads to a failure to detect the bird in the target detection algorithm correctly. Therefore, aiming at fine-grained bird recognition and detection, this paper proposes a bird recognition method based on improved YOLOV5 [29] via graph pyramid attention. This method is divided into three parts. Firstly, using a backbone network based on the CSP structure and cross-stage attention method, the CSP structure can streamline the model while attention can extract fine-grained features. Then, the graph pyramid structure is used to further extract features of different scales and for fine-grained recognition; finally, the optimized YOLOV5 architecture with Soft-NMS strategy is used for fine-grained image detection of various birds in complex natural environments.

## 2. Related Works

### 2.1. Traditional Bird Identification Dataset and Methods

Bird image recognition methods are mainly divided into machine learning and deep learning methods. The traditional recognition algorithm is based on manual extraction, feature formation, and a cascade feature classifier, and it is realized by shallow trainable architecture. A dataset is the basis of research. Many datasets for bird identification research have been published on the Internet. The BIRDS 400 bird dataset contains datasets of 400 birds, 58,388 training images, 2000 test images, and 2000 verification images. There is only one bird in each picture. All images are 224 × 224 × 3 color images in jpg format. The California Institute of Technology proposed the CUB200-2011 dataset in 2010 [30], the benchmark image dataset for fine-grained classification and recognition research. There are 11,788 bird images in this dataset, including 200 bird subcategories. The dataset is divided into training and testing, and the number of images is 5994 and 5794, respectively. Each image provides image label information, bird bounding box information, essential part information of birds, and attribute information. The Birdsnap dataset [31] contains pictures of 500 common birds in North America, with the number of pictures of each bird being about 100, for a total of 49,829 photos.

The NABirds V1 dataset is a collection of 48,000 annotated images of 400 species of birds common in North America [32]. There are more than 100 photos of each species, including individual annotations of males, females, and larvae. In the early days, people needed artificial feature designs for feature extraction, such as local binary patterns [33] and directional gradient histograms [34]. This method must first determine the features and parts to be extracted, for example, the bird’s outline, color, and texture, and 15 significant details of the bird’s mouth, feet, wings, and neck [35].

Huang et al. [36] use a graphical model considering significance to classify and annotate fine-grained bird species. The method first divides the image into several regions. Then, based on the regional and patch-level characteristics, GMS is used for classification and labeling. Finally, SVM is used as a classifier, and GMS is used for image classification. Berg et al. [37] proposed an algorithm for feature coding of local areas, automatically searching for information that plays a crucial role in image classification. However, this method requires high positioning accuracy of critical points, so only a 73.3% recognition accuracy can be obtained on CUB200-2011. These methods are suitable for simple images and a small amount of image data, but they perform poorly for images with complex backgrounds, such as the presence of occlusion or bad weather [38], and often fail to achieve the expected classification accuracy, and the generalization ability of the models are poor.

### 2.2. Bird Identification via Deep Learning Migration Technology

Thanks to the rapid development of deep learning, target recognition and classification technology have made remarkable progress. In recent years, networks, e.g., AlexNet [39], VGG [40], ResNet [41], GoogleNet [42], and DenseNet [43], have been launched one after another, which shows that convolutional neural networks have a compelling feature learning ability in image recognition. Therefore, many researchers also apply deep learning technology to classify and detect birds. In 2013, Zhang et al. [44] proposed a fine-grained classification model, R-CNN, based on parts. In this method, the depth convolution feature based on the bottom-up area suggestion calculation is used to overcome the labeling problem of the assumed bounding box in the attitude normalization process. The bounding box is modified and fine-tuned by learning the whole object and the component detector, and the geometric constraints between them are forcibly learned. Finally, a classification accuracy of 76.37% is achieved by implementing feature cascade fusion classification through AlexNet. Donahue et al. [45] analyzed the use of in-depth features in the semi-supervised multi-task framework, found that such features are superior to the most advanced feature methods based on complex multi-core learning technology and traditional manual engineering, and named them De CAF, which proves that convolutional neural network (CNN) features have more robust semantic information and better performance than artificial features. Since then, almost all bird image recognition algorithms have turned to CNN features.

Because of the apparent fine-grained problem in birds, it is also essential for research content to use the fine-grained method to identify birds. Huang et al. [46] proposed a PS-CNN method based on a partially laminated CNN structure, which, based on component-based R-CNN, modeled the subtle differences between components and objects to achieve accurate positioning of multiple target discrimination parts. By adopting a set of sharing strategies in calculating multiple object parts, the classification accuracy was 76%. Song et al. [47] introduced the feature enhancement and suppression module, which enabled the network to mine other potential features when learning the most significant features. The network could understand features of different significant levels through multilevel operations, which improved the recognition performance. Ji et al. [48] used a convolutional binary tree network based on the attention method to recognize fine-grained images. To calculate the calculation path from root to leaf in each tree, a convolution operation was added to the edge of the tree, so the final decision calculation was the synthesis of leaf node prediction.

### 2.3. Image Recognition Based on Graph Method

The graph neural network (GNN) is a new deep learning framework that has appeared in recent years. This framework can directly learn the graph structure data representing different local areas. Therefore, it is of tremendous research significance to use the advantages of graph neural networks in analyzing interpretable features of non-European data to understand the internal relations among various fine-grained features. With the development of graph neural network theory, graph neural networks have also derived many different network structures, such as the graph convolution neural network (GCN) [49] based on the spectral domain and spatial domain, graph neural network [50] based on gating, graph attention network [51] based on the attention method, graph spatiotemporal network [52] based on time and space, etc.

Zhao et al. [53] used a graph-based high-order relationship discovery method to learn the implicit connection between different areas. This method established a feature database through semantic and location perception. Then, using the semantic grouping strategy of a graph, the features of high-dimensional data are mapped to low-dimensional space, and the learning parameters are reduced. Lin et al. [54] constructed a graph convolution network method for weakly supervised fine-grained image classification based on correlation learning. This method learns the implicit relationship between different regions from the network transmission to fully mine and utilize the context relationship between other discrimination regions, thus improving the network’s recognition ability. Wang et al. [55] proposed a cross graph propagation sub-network to learn regional correlation and then weighted and aggregated other regions in a cross way to enhance each part. Chen et al. [56] used a fine-grained graph network method to represent the learning framework based on knowledge graph embedding. Firstly, this method builds a rich visual concept library as a knowledge map. Then, the gate graph neural network generates knowledge representation to realize fine-grained image recognition. Wang et al. [57] studied the confidence of graph neural networks. By applying the confidence correction structure to the graph convolution network, the faith of the graph convolution network was improved, and the classification ability of the graph was also enhanced.

## 3. Methods and Materials

**Figure 1:**
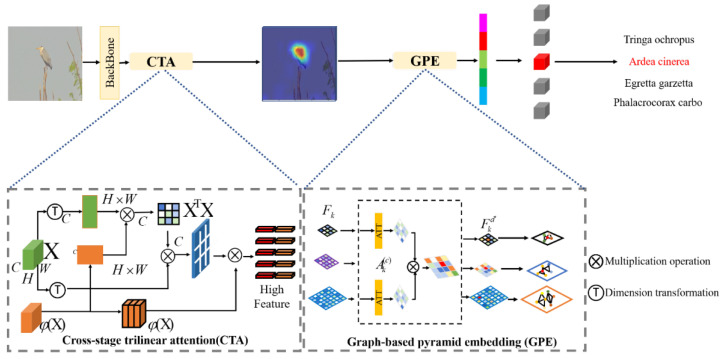
The GPA-Net framework based on a graph attention pyramid. We generate a library through the cross-stage attention module (CTA) and use feature maps of different levels to form an attention pyramid. Then, the pyramid layer is embedded into features by the graph method to form a graph attention pyramid network (GPE). Finally, multiple features are connected to obtain attention representation.

The overall framework of this paper is mainly composed of three parts: data pre-processing, a fine-grained classifier, and an optimized detector. The framework structure is shown in Figure 1. Among them, the data pre-processing component introduces various strategies used to process the data. Part of the classifier (defined as GPA-Net) uses the graph-based pyramid attention method, which is mainly composed of a CSP-based backbone network and pyramid attention structure [58]. The Yolov5 detector is used as the detector in the detection part, and the Soft-NMS mechanism is used in the detection part to enhance the detection results.

The proposed fine-grained classifier, GPA-Net, mainly consists of the backbone structure based on the CSP structure and the fine-grained feature learning structure of graph pyramid attention, as shown in Figure 1. The specific functions of each part are as follows.

### 3.1. Lightweight Backbone Network

**Figure 2:**
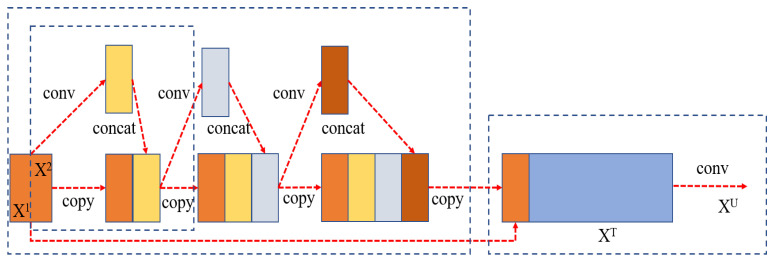
CSP network structure.

The improved CSP backbone network is used for processing as shown in Figure 2. CSP is a new variant of the ResNet network series. This structure prevents too-much-repeated gradient information by cutting off the gradient stream, strengthens CNN’s learning ability, eliminates the computational bottleneck, and effectively reduces the memory cost. The staging module of the basic CSPNet includes a primary branch and a spanning branch, and the characteristics of the two branches are spliced at the end of each stage.

The phase module of the basic CSPNet includes the basic branch and the spanning branch, and the features of the two branches are spliced at the end of each phase, as shown in Figure 2. The input of each stage first goes through two 1 × 1 convolutions. The calculation process is as follows:

(1) $$X_{Base}=F_{base}(f_{1\times 1}(X))$$

where $f_{1\times 1}$ represents 1 × 1 convolution and $F_{base}$ is a combination of n basic modules. Then, two groups of features of the basic branch and the generated branch are spliced together, and the information exchange between the two groups of features is increased by channel shuffling [48]. Finally, the output of each stage is obtained by the down-sampling module. The calculation process is as follows.

(2) $$\tilde{X}=F_{down}(S(X_{Base},X_{Cross}))$$

where $X_{Base}$ represents the output of the basic branch, $X_{Cross}$ represents the output across the branch, S represents the channel shuffling, and $F_{down}$ represents the down-sampling.

### 3.2. Cross-Stage Three-Linear Attention Fine-Grained Feature Learning Module (CTA)

**Figure 3:**
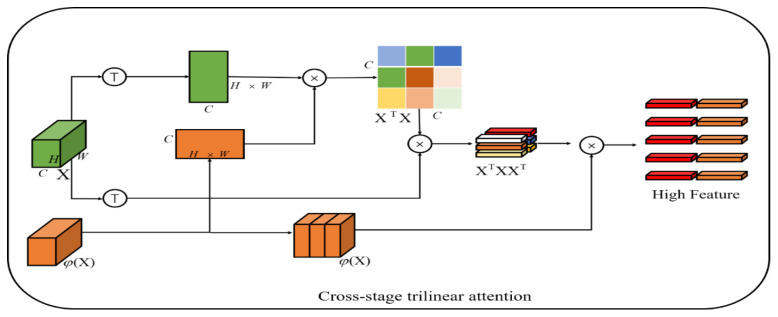
Cross-stage three-linear attention fine-grained feature learning module (CTA).

To mine plentiful features suitable for fine-grained image classification, inspired by [46], we propose a fine-grained feature learning module based on cross-stage trilinear attention as shown in Figure 3.

Through the CSP backbone network, we can obtain a feature map $\tilde{X}\in {\mathbb{R}}^{W\times H\times C}$, of which W,H represent the feature map dimensions and the channel number of the feature map, respectively. Generally, the global average pool (GAP) or global maximum pool (GMP) is used to learn the final features $\tilde{X}$. One of the common problems with average or maximum pooling is that the interactive information between different semantic channels cannot be fully utilized. 

Therefore, a common method is to use bilinear spatial attention. The second-order matrix $F^{a}$ of each position (i,j) is classified by merging itself $X_{A}\in {\mathbb{R}}^{WH\times C_{A}}$ with another CNN stream $X_{B}\in {\mathbb{R}}^{WH\times C_{B}}$, and then the final feature is used in the final category through a fully connected (fc) layer.

(3) $$F^{a}=\frac{1}{WH}\sum_{i=1}^{W}\sum_{j=1}^{H}v({\left(X_{A}\right)}_{i,j}^{⊤}{\left(X_{B}\right)}_{i,j})$$

(4) $$F^{b}=W\cdot F^{a}+b$$

where $v:{\mathbb{R}}^{C_{A}\times C_{B}}\to {\mathbb{R}}^{C_{A}C_{B}\times 1}$ represents the vectorization of the second-order matrix and $W\in {\mathbb{R}}^{C_{A}C_{B}\times N_{cls}}$ is the learnable weight of the FC layer. Although abundant features are obtained through bilinear pooling, these high-dimensional features are easily optimized with poor usability. Therefore, in the method of trilinear attention, the cross-channel relationship is regarded as the attention diagram generated by the same feature map $X^{T}X\in {\mathbb{R}}^{C\times C}$ by non-local operation, and then the channel-perceived attention map gives different annotations to the original features to produce the third-order result $S(X^{T}X)X^{T}\in {\mathbb{R}}^{WH\times C}$, where S represents SoftMax normalization.

However, a disadvantage of the cross-channel relationship is that it ignores the learning between multi-scale features. Because different network layers have different scales of receptive fields, the later network layers have larger receptive fields. Inspired by [47], we use features φ(X) from the next layer of the same stage of the network to conduct cross-layer semantic learning of cross-channels (see Figure 3), which can be expressed as:

(5) $$F^{c}=N(M(\frac{1}{WH}\sum_{i=1}^{WH}(X^{⊤}\varphi (X))))\in {\mathbb{R}}^{C\times HW}$$

where φ(X) is the output of one layer after the same stage of CSP, N denotes SoftMax normalization, and $M(x)=sign(x)x^{-1/2}$ denotes moment function normalization. Similar to trilinear attention, in order to make the feature map more consistent and enhance robustness, the spatial relationship is further integrated into the feature map by $F^{c}$ dot multiplication of $F^{c}$ and φ(X), so a cross-stage trilinear attention map is obtained. The attention map can be expressed as:

(6) $$F^{d}=N(M(F^{c^{⊤}}\varphi (X)))\in {\mathbb{R}}^{C\times HW}$$

Therefore, this paper constructs an attention map $F^{d}$, and each channel of the map $F^{d}$ represents an attention map $F_{i}^{d}\in {\mathbb{R}}^{W\times H}$. The pyramid method is used to learn objects with different scales in images:

(7) $$F_{k}^{d^{\prime }}=\mathrm{CTA}({F^{\prime }}_{k})$$

where CTA denotes cross-stage trilinear attention and ${F^{\prime }}_{k}$ represents the characteristics of the k level of the pyramid. The values of *k* are 2, 3, and 4, representing the second, third, and fourth stages of the network, respectively.

### 3.3. Graph-Based High-Order Feature Embedding (GFE)

Multi-scale fine-grained feature libraries can be generated through the cross-stage attention modules in Section 3.3. Inspired by [47], each element of these feature libraries can be regarded as a graph node, and then the adjacency matrix score of the node graph neural network is used to embed these features.

Firstly, every element in attention $F^{d}=\{f_{1},\cdots ,f_{C_{N}}\}$ is regarded as a graph node, and each graph node shares a large amount of information, so we can aggregate these nodes in the following ways.

(8) $$A_{i,j}=\frac{\tau {\left(f_{i}\right)}^{T}\cdot \tau (f_{j})}{\left||\tau (f_{i})||||\tau (f_{j})|\right|}$$

where τ is the convolution used for dimension transformation and represents $A_{i,j}$, the adjacency matrix score of nodes i and j. The adjacency matrix is $\tilde{A}=A+I$, where $I\in {\mathbb{R}}^{C_{1}\times C_{1}}$ is the identity matrix.

Through similarity aggregation in this way, each node is updated as follows:

(9) G0=ReLu(D˜−12A˜D˜−12KdWd)

where $W_{d}$ is the graph node weight of $d_{h}$ learnable dimension and ${\tilde{D}}_{d}=\sum {}_{j}{\tilde{A}}_{i,j}^{d}$ is the diagonal matrix to be normalized in nodes. $K_{d}$ represents the matrix form of feature bank $\kappa_{d}$.

Similarly, the features $F_{k}^{d^{\prime }}$ in the pyramid structure also have embedded features $G_{k}^{d^{\prime }}$ through similar operations, as shown below:

(10) Gk=ReLu(D˜k−12A˜kD˜−12k KkWk)

where $W_{k}$ is the graph node weight of learnable $d_{h}$ dimension, ${\tilde{D}}_{k}=\sum {}_{j}{\tilde{A}}_{i,j}^{k}$ is the diagonal matrix to be normalized, and $K_{k}$ represents the matrix form of feature library $\kappa_{k}$.

Through the above graph propagation structure, we obtain the embedded features $G=\{G_{0,}G_{1,}\cdots ,G_{k}\}$ of a multilevel structure. Considering that the features of different levels play different roles, we use an adaptive attention mechanism $I=\{I_{0},I_{1},\cdots ,I_{k}\}$ to learn the importance between them, as shown below:

(11) I=att(G)

where att denotes attention, and its specific generation mode is as follows. There is a node $g_{m}^{i}$, where 0≤m≤k and i denotes the i-th point. Secondly, we use nonlinear transformation to transform the embedding and then use an attention vector η to obtain the attention value, as shown below:

(12) $$\eta_{m}^{i}=\tanh(W_{m}\cdot {\left(g_{m}^{i}\right)}^{T}+b_{m})$$

where tanh is a nonlinear function, $W_{m}$ is a learnable parameter weight, and $b_{m}$ is a bias. Through a similar operation, we can obtain all the embedded $G=\{G_{0,}G_{1,}\cdots ,G_{k}\}$ attention values and normalize them with the SoftMax function:

(13) $$I_{m}^{i}=softmax(\eta_{m}^{i})=\frac{\exp(\eta_{m}^{i})}{\sum_{m=0}^{k}\eta_{m}^{i}}$$

The larger the $I_{m}^{i}$, the more important the embedding. For all n nodes. The attention vector $I=[I_{0},\cdots ,I_{k}]$ can be obtained at last, which is selected to distinguish the importance of different levels of features through attention adaptation. The final result score is as follows:

(14) $$C=\sum_{m=0}^{k}I_{m}soft\max(G_{m})$$

### 3.4. YOLOV5 Detector via Soft-NMS Optimization

**Figure 4:**
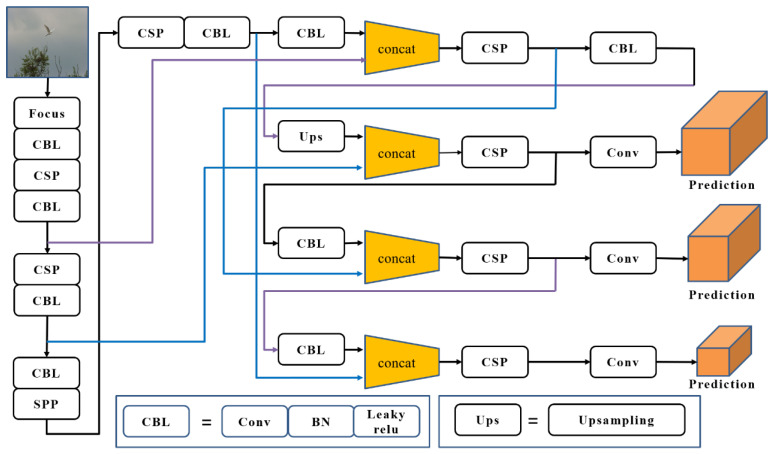
Optimized YOLOV5 detector structure.

The target detection task classifies and locates the objects in the image, generates a detection frame for the objects in the image, and generates corresponding confidence. YOLOV5 is used as the detector in this paper (Figure 4). When the model detects the detection frame of the object in the predicted image, multiple detection frames are generated for the same object, and each detection frame has a corresponding score. Having too many detection frames reduces the detection accuracy. Therefore, the NMS method is used to process the detection frame and obtain the final detection result. So far, this method is still a popular target detection algorithm and can effectively improve the recall rate of detection. Non-maximum suppression (NMS) is an integral part of the target detection model. The detection box is screened to reduce the repetition rate and improve the recall rate of detection.

The basic flow of this method is to sort the generated detection frames according to the scores, take out the detection frame M with the highest score, compare the rest with the selected detection frames, and suppress the detection frames whose overlap is higher than the threshold. This process is repeatedly applied to the rest of the detection frames until they are compared with the detection frames below it. The traditional non-maximum suppression method directly zeroes the score whose overlap ratio with the adjacent detection frame is greater than the threshold so that when two detected objects are close, the traditional non-maximum suppression method deletes the detection frame with the lower score, resulting in missed detection, which leads to the decrease in the accuracy of the detection results.

(15) $$S_{i}=\left\{\begin{array}{c} S_{i},\;IOU(M,b_{i})<N_{t}\\ 0,\;IOU(M,b_{i})\geq N_{t} \end{array}\right.$$

The traditional non-maximum suppression algorithm has the disadvantage of missing overlapping objects, so we adopt a new soft non-maximum suppression algorithm, which is improved on a conventional basis. In this algorithm, when the overlap of two detection frames is more significant than the threshold, it is deleted and improved, but an attenuation function is set. When the overlap between the rest of the detection frames and *M* is greater than the threshold, a very low weight is given. If only a tiny part of the detection frames overlap, it does not affect the detection results. Soft-NMS does not need additional training; it directly trains the model from end to end without increasing the model parameters.

When there is a high overlap with the detection box *M*, it is set to fractional attenuation instead of directly setting it to zero. The higher the overlap, the more serious the corresponding fractional attenuation. Therefore, the traditional non-maximum suppression method is improved, as shown in Formula (15).

(16) $$S_{i}=\left\{\begin{array}{c} S_{i},\;\;\;\;\;\;\;\;IOU(M,b_{i})<N_{t}\\ S_{i}(1-IOU(M,b_{i})),\;IOU(M,b_{i})\geq N_{t} \end{array}\right.$$

By improving the traditional non-maximum suppression method, a fractional attenuation method is proposed to solve the problem of missing detection when the detection frames overlap. It is verified on two existing public detection datasets. Through the experimental analysis, it is found that the improved non-maximum suppression method can identify overlapping objects and improve the detection accuracy of the model. The Soft-NMS method only improves the traditional non-maximum suppression algorithm, which does not increase the parameters of model operation and affects the detection speed of the model. It is easy to implement and can be embedded in any detection model.

### 3.5. Loss Function

In the training process, the cross-entropy loss is used as the loss function, and in order to reduce the risk of over-fitting, it is also applied to label smoothing technology, and the smoothed new label is used to replace the original label:

(17) $$y^{\prime }=(1-\varepsilon )\tilde{y}+\varepsilon \mu$$

where $\tilde{y}$ represents the sample label, ε is the smoothing factor, and u is a fraction of the category. Using label smoothing can make the classification probability result of the SoftMax activation function close to the correct classification, restrain the output difference between positive and negative samples, and make the network have better generalization ability.

The *CIOU* loss function of the detector used in YOLOV5 is to measure the loss of rectangular frames. The specific formula is as follows:

(18) $$S_{1}=(\min(x_{p2},x_{l2})-\max(x_{p1},x_{l1}))\times (\min(y_{p2},y_{l2})-\max(y_{p1},y_{l1}))$$

(19) $$S_{2}=(x_{p2}-x_{p1})\times (y_{p2}-y_{p1})+(x_{l2}-x_{l1})\times (y_{l2}-y_{l1})-S_{1}$$

(20) $$IOU=\frac{S_{1}}{S_{2}}$$

(21) $$CLOU=IOU-\frac{\rho^{2}}{c^{2}}-\alpha v$$

(22) $$v=\frac{4}{\pi^{2}}{\left(\arctan\frac{w_{l}}{h_{l}}-\arctan\frac{w_{p}}{h_{p}}\right)}^{2}=\frac{4}{\pi^{2}}{\left(\arctan\frac{x_{l2}-x_{l1}}{y_{l2}-y_{l1}}-\arctan\frac{x_{p2}-x_{p1}}{y_{p2}-y_{p1}}\right)}^{2}$$

(23) $$\alpha =\frac{v}{1-IOU+v}$$

where ρ is the distance between the center points of box A and box B, c is the diagonal length of the minimum bounding rectangle of box A and box B, v is the width–height similarity ratio of frame A and frame B, and α is the influence factor of v. The calculation formula of *CIOU* loss can be obtained from the above:

(24) $$loss_{CIOU}=1-CIOU$$

*CIOU* considers the distance between the target and the anchor, the overlap rate, the scale, and the penalty term so that the target box regression becomes more stable, and there is no divergence or other problems in the training process such as *IOU* and *GIOU*. The penalty factor considers the aspect ratio of the prediction frame and the aspect ratio of the target frame.

## 4. Experiment and Result

### 4.1. Implementation Details

In this experiment, we pre-trained our model on the ImageNet dataset. Pre-processing adopts random cutting and random turning. The figure size was resized to 448 × 448, the batch size was set to 128, training times were 150, and SGD was used as an optimizer. Meanwhile, the cosine annealing strategy was used to attenuate the learning rate, and the attenuation period was 20. The final experiment was conducted on 8 Nvidia P40 graphics cards.

In the classification experiment, we adopted two indexes: the accuracy rate (ACC) and the parameter size of the model parameters. The accuracy calculation formula is as follows:

(25) $$ACC=\frac{TP+TN}{TP+TN+FP+FN}$$

Here, *TP* represents true positive, *TN* represents true negative, *FP* represents false positive, and *FP* represents false negative. In the testing experiment, the precision (*P*), recall (*P*), and average accuracy are used as evaluation indexes, and the calculation formula is as follows:

(26) $$P=\frac{TP}{TP+FP}$$

(27) $$R=\frac{TP}{TP+FN}$$

(28) $$\mathrm{AP}=\int_{0}^{1}\left[P(R)dR\right]$$

where *P* denotes precision and *R* denotes recall. Different evaluation indexes can not only evaluate the model in all aspects but also further prove the superiority and practicability of the proposed method.

### 4.2. Bird Classification Results

To verify the performance of the fine-grained methods, we used two fine-grained datasets for experiments and selected some open-source algorithms for performance comparison. 

Dataset (1) CUB-200-2011 [35] contains 5994 images in the training process and 5794 images in the test process; (2) Bird-400 contains 58,388 images in training datasets and 2000 images in test sets. The comparison index selected the commonly used accuracy index of image classification. Meanwhile, the possibility of the fine-grained method is discussed according to a comparison of the parameters of different models as shown in Table 1.

**Figure 5:**
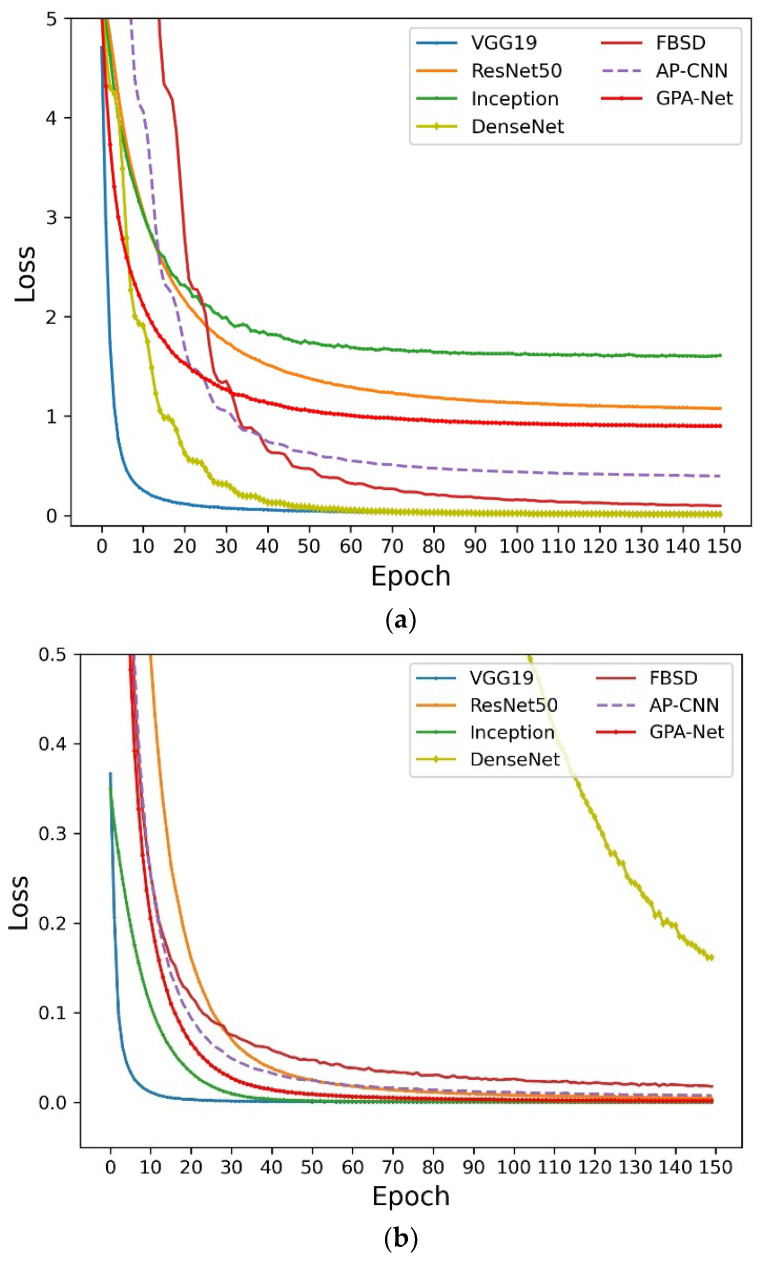

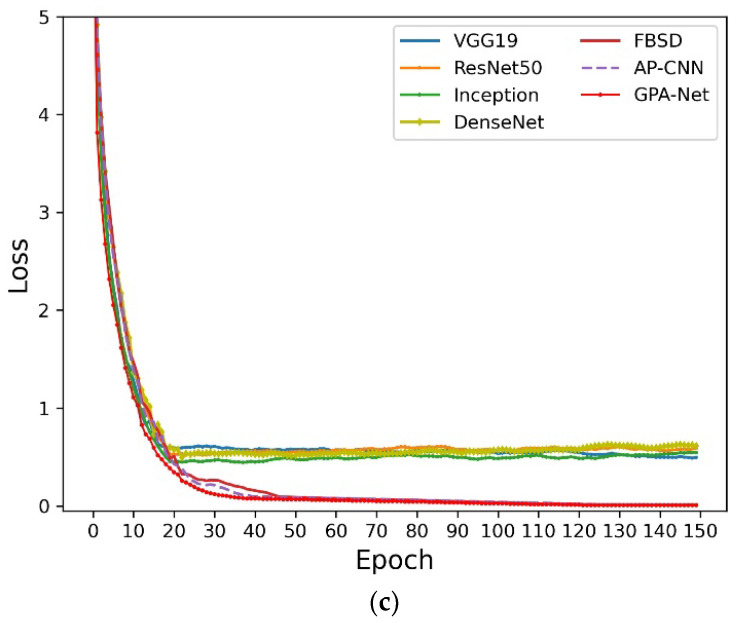
(**a**) The loss curves of each model on CUB-200-2011. (**b**) The loss curves of each model on Bird-400. (**c**) The loss curves of each model on AF-bird50.

From the classification results shown in Table 1, we can see that the results of GPA-Net are better than those of coarse-grained methods, e.g., VGG, ResNet, GoogleNet, and DenseNet, which shows that the GPA-Net can learn more new information and has greater vital feature extraction ability. Meanwhile, compared with some fine-grained methods, our method has more advantages. On the CUB-200-2011 dataset, GPA-Net has achieved the best results, with an accuracy rate of 89.6%. Compared with other fine-grained methods, the results on the Bird-400 dataset are not much different. This is because the Bird-400 dataset is more direct and has less interference than other datasets. Comparatively speaking, our method is better at feature extraction in complex backgrounds. The results show that FBSD and AP-CNN benefit from the simple backgrounds on the Bird-400 dataset. However, our method has more advantages in datasets with a more complex background, e.g., CUB2011 and AF-bird50. In terms of the parameters of the model, compared with the coarse-grained method, our model is slightly larger than the standard coarse-grained method, but its accuracy is better. Compared with other fine-grained methods, our model is lighter, which shows that our model has advantages in practical application. We drew a comparison chart of the loss change during model training to further explore and evaluate the model’s performance, as shown in Figure 5.

**Figure 6:**
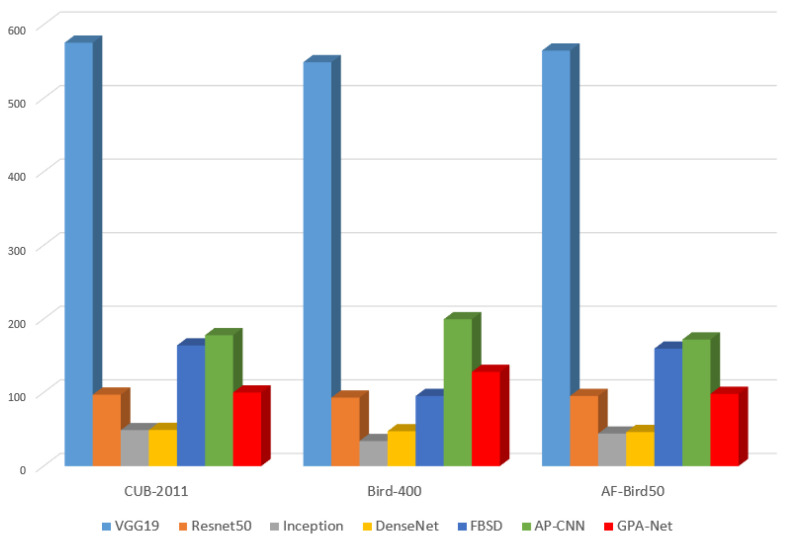
Parameter comparison chart of different models.

The downward trend of the loss is shown in Figure 5. Obviously, we know that GPA-Net can drop rapidly in the training process and reach a stable state later, indicating that the model has strong stability in the training process. Meanwhile, it can be seen that GPA-Net converges faster than other models in the early stage, and the loss value is lower after reaching the steady state, which shows that GPA-Net has better convergence and can learn more. We use a contrast histogram to analyze the parameter amounts of different models on three datasets, as shown in Figure 6.

The comparison results of model parameters are shown in Figure 6. The parameters of the GPA-Net model are slightly larger than those of ResNet50, Inception, and DenseNet because the attention module added to the GPA-Net model occupies a part of the parameters. At the same time, the parameters of GPA-Net are much smaller than those of other fine-grained algorithms. This is because the backbone network of our model uses a CSP structure, and the graph embedding method is used to reduce the parameters further, showing that our model is more applicable in practical applications.

### 4.3. Bird Detection Results

Although there are many bird species in existing public bird databases, they mainly rely on the Internet to crawl data. On the one hand, the scene of each image is an ideal shooting environment (many pictures are provided for photography enthusiasts), and different pictures have significant picture quality. On the other hand, these datasets are mainly collected for the purpose of bird image classification. A picture often has only a small sample of the same bird, which cannot solve the picture detection tasks that need to identify multiple samples of different birds appearing in the same photo. When a dataset is used to conduct deep learning network training, it cannot solve the real-scene application of actual environmental protection. In the remote monitoring system of environmental protection, the background of an effective picture is often very complicated. Many samples of different types of birds appear randomly together, and the proportion of each bird occupying the entire picture is usually small. There are also challenges such as mutual obstruction between goals and backgrounds or the appearance similarity of different birds. Therefore, deep learning models only relying on public datasets cannot effectively solve the image recognition task in practical applications.

Therefore, in order to solve the challenge of bird detection in actual environmental protection applications, we took the main bird species living in river and lake environments in Beijing, China, as research objects and built a new dataset, AF-bird50, for application demonstration experiments. AF-bird50 contains over 10,000 images, covering 50 different species. Our dataset comes from photos taken in three stations—Miyun Reservoir Station 1, Guanting Station 1, and Liudu Station in Fangshan District of Beijing—and these stations were set up starting in March 2022. All images were mainly collected through cameras, and sensors were also applied to obtain bird pictures in real scenes by ourselves. We tested the AF-bird50 dataset and selected SSD-300, Faster-R-CNN, and YOLOV3 as the comparison models. We divided this dataset into a ratio of 3:7; thus, there are 7000 training sets and 3000 test sets. The experimental results can be seen in Table 2.

**Figure 7:**
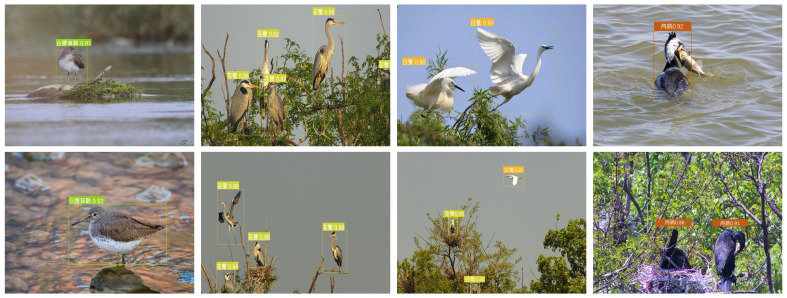
The detaching results obtained by optimized YOLOV5 model.

From Table 2, we can see that compared with other target detection models, e.g., Faster-R-CNN, YOLOV3, etc., the optimized YOLOV5 achieves the highest results for different indicators, e.g., precision rate and recall rate. Thereby, the optimized YOLOV5 based on the proposed GPA-Net is suitable for accurate bird detection. Some bird detection results are shown in Figure 7. As shown in the picture, the species of these wild birds include sandpiper (scientific name “Tringa ochropus”, Chinese name “白腰草鹬”), heron (scientific name “Ardea cinerea”, Chinese name “苍鹭”), egret (scientific name “Egretta garzetta”, Chinese name “白鹭”), cormorant (scientific name “Phalacrocorax carbo”, Chinese name “鸬鹚”), etc. All pictures were obtained through remote monitoring cameras in natural environments such as lakes and rivers.

**Figure 8:**
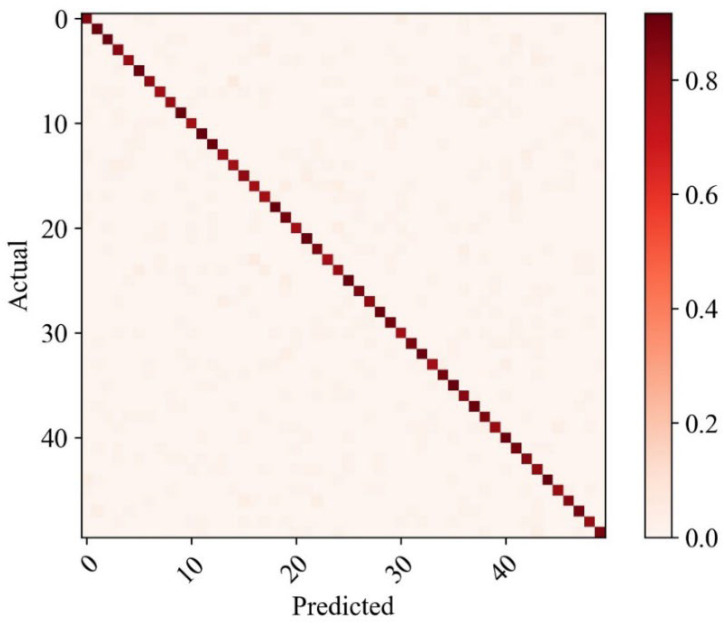
Confusion matrix on AF-bird50.

It can be seen from Figure 7 that the network can detect the target very accurately for the sample with only a single target and simple background in the image. On the other hand, in the presence of interference factors such as multiple targets, different poses, and background occlusion in the sample images, the network still achieves a sound effect, which indicates that the Soft-NMS mechanism can improve the performance of the network well and further demonstrates that the optimized YOLOV5 network can be used in actual bird recognition tasks. Furthermore, we draw a confusion matrix of the proposed detector’s accuracy to visualize the model’s performance, as shown in Figure 8.

The result of this confusion matrix is shown in Figure 8. It can be seen that the recognition rate of all birds is almost above 90%, which indicates that the model has high accuracy. At the same time, it can be seen that among the samples with model recognition errors, none identify a large number of samples as the existence of another bird, which indicates that the model has high fault tolerance. The results of recognition errors are mainly caused by vague pictures and severe occlusion, which lead to the model learning less than the required features.

## 5. Discussion

To verify the actual function of each module in the model, we set up an ablation experiment to analyze the model on the CUB-200-2011 dataset. The ablation experiment was executed for further analysis of module functions. When only relying on the CSP backbone network, the base model achieves an accuracy of 85.1%. After using the CTA module, the accuracy is improved by 0.8%, achieving a result of 86.3%. In contrast, after only using the GPE module, the accuracy of the model is up to 87.2%, which is 2.1% higher than that of the backbone network. When the CTA and GPE modules are applied to improve performance at the same time, the entire model can achieve an 89.6% accuracy, which is the highest result. This shows that the pyramid structure can help the model learn more fine-grained features and improve its fine-grained identification ability.

In particular, the CSP is used as the backbone of our method to learn the shallow feature information of images. Based on these shallow features in different stages, we can further learn high-order feature information with discriminant information. Meanwhile, the CTA attention module is used to mine the learned features further. The pyramid structure is used to learn the features of different scales for smaller targets. The graph structure is used to learn parts of different scales, and attention adaptive learning is used to understand the importance of different levels. The ablation experiment shows that the CTA module can strengthen feature learning and improve accuracy by 0.8%.

**Figure 9:**
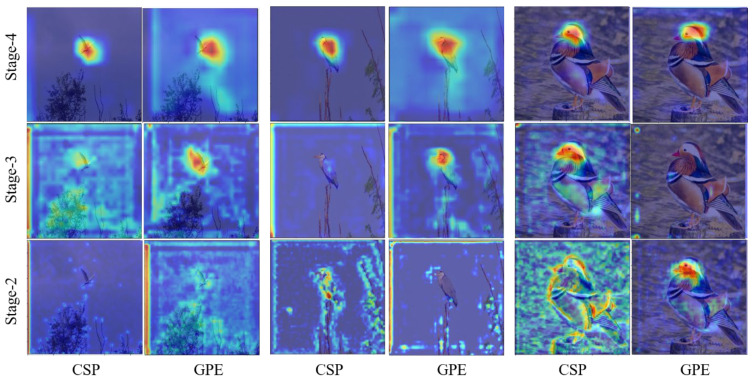
Heat map visualization of attention distribution for GPE module.

Meanwhile, the GPE module can adaptively distinguish the importance of different levels, reduce the influence of redundant features, and improve recognition accuracy. The analysis of the ablation experiment proves that our network can learn different regional features with discrimination information and improve the accuracy of fine-grained recognition. For visual analysis, we use a heat map of attention distribution to visualize the GPE module and explore its essential role. The visualization results of Stage2 to Stage4 for three natural samples are shown in Figure 9.

As shown in above Figure 9, if only the last layer of the backbone network is used as the classification feature, the network misses a large amount of other useful information, and other supplementary information is learned for classification by using the pyramid structure. Meanwhile, to prevent the features at the bottom stage from bringing too much noise, the attention mechanism is added to adaptively judge the importance of these features at different levels.

## 6. Conclusions

The avian species are essential to protecting the ecological environment and maintaining biodiversity change. This paper proposes a fine-grained bird recognition method based on a graph attention pyramid to solve the fine-grained problem in bird image recognition. Firstly, based on the CSP baseline network, the fine-grained feature of cross-stage attention module learning is designed. At the same time, the graphic pyramid structure is introduced to learn the multi-scale features for understanding the context information between different local parts and then building a novel fine-grained classifier GPA-Net. Finally, an optimized YOLOV5 with Soft-NMS strategy is designed as the detector composition, improving the detection capability for small targets. Massive experiments demonstrated that the performance of the proposed method for fine-grained bird identification is better than other comparative models. The accuracy rates of GPA-Net on CUB-200-2011, Bird-400, and AF-bird50 datasets are 89.6%, 99.3%, and 95.4%, respectively, reaching the optimal results. Additionally, the optimized YOLOV5 detector based on GPA-Net has the advantages of smaller identification error and lighter model parameters. This shows that the proposed method is more suitable for natural bird image recognition in complex scenes.

Optimizing the model structure and improving model performance will be our work in the future. At the same time, improving the model’s generalization ability is also an essential part of future work. In the future, we will explore the application potential of these methods in other fields, such as motion estimation [62], modeling optimization [63], and temporal prediction [64], etc.

## Figures and Tables

**Table 1:** The classification results of three datasets.

		CUB-200-2011		Bird-400		AF-Bird50	
Methods		ACC (%)	Parameter (M)	ACC (%)	Parameter (M)	ACC (%)	Parameter (M)
Coarse-grained	VGG19 [42]	76.4	575.5	95.4	549	80.1	564.8
	ResNet50 [43]	85.2	97.1	96.8	93.1	87.2	95.4
	Inception [44]	85.9	48.9	97.6	34.1	88.3	44.2
	DenseNet [45]	86.1	49.2	97.4	47.4	90.1	46.3
Fine-grained	FBSD [49]	89.2	164	99.2	95.3	94.3	159.7
	AP-CNN [59]	88.1	178	99.3	199.6	94.6	172.1
	GPA-Net	89.6	100	99.3	128	95.4	98.2

**Table 2:** Bird detection results on AF-bird50.

Method	P(%)	R(%)	AP(%)
SSD300 [60]	77.38	79.47	78.43
Faster-R-CNN [61]	80.01	81.69	80.52
YOLOV3 [31]	85.89	87.92	86.91
Optimized YOLOV5	88.43	90.64	89.37

## Data Availability

Datasets contained within the article are available at the following links. CUB-200-2011: https://www.vision.caltech.edu/datasets/cub_200_2011 (accessed on 19 January 2023). Bird-400: https://www.kaggle.com/datasets/gpiosenka/100-bird-species (accessed on 19 January 2023).
